# Messenger RNA biomarkers of Bovine Respiratory Syncytial Virus infection in the whole blood of dairy calves

**DOI:** 10.1038/s41598-021-88878-1

**Published:** 2021-04-30

**Authors:** Dayle Johnston, Bernadette Earley, Matthew S. McCabe, JaeWoo Kim, Jeremy F. Taylor, Ken Lemon, Catherine Duffy, Michael McMenamy, S. Louise Cosby, Sinead M. Waters

**Affiliations:** 1 Animal and Bioscience Research Department, Animal and Grassland Research and Innovation Centre, Teagasc, Grange, Co., Meath, Ireland; 2 Division of Animal Sciences, University of Missouri, Columbia, MO USA; 3 Veterinary Sciences Division, Agri-Food and Biosciences Institute, Stormont, Belfast, Northern Ireland

**Keywords:** Immunology, Molecular biology, Systems biology, Biomarkers

## Abstract

Bovine Respiratory Syncytial Virus (BRSV) is a primary viral cause of Bovine Respiratory Disease (BRD) in young calves, which is responsible for substantial morbidity and mortality. Infection with BRSV induces global gene expression changes in respiratory tissues. If these changes are observed in tissues which are more accessible in live animals, such as whole blood, they may be used as biomarkers for diagnosis of the disease. Therefore, the objective of the current study was to elucidate the whole blood transcriptomic response of dairy calves to an experimental challenge with BRSV. Calves (Holstein–Friesian) were either administered BRSV inoculate (10^3.5^ TCID_50_/ml × 15 ml) (n = 12) or sterile phosphate buffered saline (n = 6). Clinical signs were scored daily and whole blood was collected in Tempus RNA tubes immediately prior to euthanasia, at day 7 post-challenge. RNA was extracted from blood and sequenced (150 bp paired-end). The sequence reads were aligned to the bovine reference genome (UMD3.1) and EdgeR was subsequently employed for differential gene expression analysis. Multidimensional scaling showed that samples from BRSV challenged and control calves segregated based on whole blood gene expression changes, despite the BRSV challenged calves only displaying mild clinical symptoms of the disease. There were 281 differentially expressed (DE) genes (*p* < 0.05, FDR < 0.1, fold change > 2) between the BRSV challenged and control calves. The top enriched KEGG pathways and gene ontology terms were associated with viral infection and included “Influenza A”, “defense response to virus”, “regulation of viral life cycle” and “innate immune response”. Highly DE genes involved in these pathways may be beneficial for the diagnosis of subclinical BRD from blood samples.

## Introduction

Bovine Respiratory Syncytial Virus (BRSV) is a leading cause of Bovine Respiratory Disease (BRD), which is responsible for significant morbidity and mortality (ranging from 5 to 11%^1^) in dairy calves globally. Morbidity rates ranging from 60 to 80% have been reported due to BRD caused by infection with BRSV and the disease symptoms can vary from sub-clinical to severe clinical signs including coughing, pyrexia, nasal discharge, anorexia and respiratory distress^2^.

Bovine Respiratory Disease is multifactorial and is influenced by environmental, husbandry and management factors, in additional to genetic predisposing factors^3^. It is caused by primary viral and bacterial members of the Bovine Respiratory Disease Complex (BRDC), including BRSV, which is an enveloped, negative sense, single stranded RNA virus (2). We^4^ and others^5,6^ have shown that BRSV infection induces substantial alterations in bovine host gene expression in the bronchial lymph node and lung tissues. If these changes in gene expression due to BRSV infection are also detected in whole blood, they could potentially be used in a molecular qPCR or sequencing based assay for the diagnosis of BRSV. Unlike lung and lymph node respiratory tissues, which are only accessible following invasive tissue biopsies or euthanasia, and therefore would not be practical for sampling for diagnostic purposes in live animals, blood is easily accessible and routinely collected in bovine clinical health assessments^7^. Additionally, differences in gene expression in whole blood have been identified in beef feedlot cattle that subsequently naturally acquire BRD and those who remain BRD free at feedlot entry^8^. These authors found that the identification of DEGs in the whole blood of calves at arrival had a clear distinction between calves that went on to develop BRD and those that were resistant to BRD^8^. Consequently, it is likely that BRSV specifically induces gene expression changes in whole blood in infected cattle and these changes may be used to identify BRSV as the causative agent of the BRD infection. Therefore, the objective of this study was to identify differentially expressed (DE) genes due to an experimental challenge with BRSV in artificially-reared dairy calves. Employing this approach, the DE genes could potentially be used in novel blood-based non-invasive diagnostic tests for the confirmation of infection with BRSV in calves.

## Methods

### Animal model

All animal studies were carried out in accordance with the UK Animals (Scientific Procedures) Act 1986 and with the approval of the Agri-Food and Biosciences Institute Northern Ireland Ethical Review Committee. This study was carried out in compliance with the ARRIVE guidelines (http://www.nc3rs.org.uk/page.asp?id=1357).

The animal model and animal sampling used in this experiment has been previously described^4^. Briefly, animals were selected from a population of 30 Holstein–Friesian bull calves (mean age ± s.d. = 120.7 ± 14.15 days) and recruited into the study based on low BRSV specific maternally derived antibody (MDA) levels and negative BRSV PCR status two weeks prior to challenge. Recruited animals were assigned to three groups (A, B and C) based on sire, age, weight and MDA. There were two treatment groups in the study; BRSV challenged where calves were challenged with BRSV (n = 12; groups B and C) and control, where calves were mock challenged with sterile phosphate buffered saline (PBS) (n = 6; group A). Groups A, B and C were housed in separate locations.

### Animal sampling

Clinical signs of BRD were recorded daily, from challenge to the day of slaughter, and scored by a veterinarian, who was blinded to the calves’ treatment status (BRSV challenged or control), using a modified version of the clinical scoring system^4^ described by^9^. On Day 0, calves were either challenged with 10^3.5^ TCID_50_/ml × 15 ml of BRSV strain SVA 274/92 inoculum (BRSV challenged calves in groups B and C) or mock challenged with 10 ml PBS (control calves in group A), by aerosol inhalation using the Omron NE-U780 nebuliser (OMRON Model: NE-U780-E S/N: 20151200182AF) fitted with a veterinary face mask (GaleMed Model: VM-2 Animal Mask size 5 22mmlD Ref: 5635 Lot: 151019)^4^.

On day 7 relative to the challenge, calves were euthanized by captive bolt. Whole blood was collected in Tempus RNA tubes immediately prior to euthanasia. The Tempus tubes were stored at  − 80 °C until analysis. The lungs were scored for lesions by a veterinarian using an AFBI standard lung scoring system^4^ which assigns the percentages of lesions present on the total lung area and on component parts of the lungs.

### RNA extraction

Total RNA was extracted using the Tempus Spin RNA Isolation Kit (Bio-Sciences LTD, Dublin, Ireland), according to the manufacturer's instructions. The quantity of the extracted RNA was determined by measuring the absorbance at 260 nm with a Nanodrop spectrophotometer (NanoDrop technologies; Wilmington, DE, USA). The Agilent 2100 Bioanalyser (Agilent Technologies Ireland Ltd; Dublin, Ireland) with the RNA 6000 Nano LabChip kit (Agilent Technologies Ireland Ltd; Dublin, Ireland) was used to examine the quality of the extracted RNA. Samples had a mean RNA Integrity Number of 9.7 (± S.D. 0.34).

### RNA-Seq library preparation and sequencing

Extracted RNA was shipped frozen at  − 80 °C on dry ice to the University of Missouri’s DNA Core Facility for RNA-Seq library preparation using the TruSeq stranded mRNA Kit (Illumina, San Diego, California, USA) and high-throughput sequencing (150 bp paired-end) on an Illumina NovaSeq 6000. All sequence data produced in this study has been deposited to NCBI GEO repository and are available through the series accession number GSE152959.

### Alignment of sequence reads to the bovine reference genome and differential gene expression analysis

The 3′ ends of the sequence reads were trimmed for Illumina adapter sequence, low quality reads (quality score < 20), ambiguous nucleotides, and poly-G artefacts resulting from the NextSeq’s two-colour chemistry, using CutAdapt (version 1.18)^10^. The quality assessment, alignment to the reference genome, data normalisation and differential gene expression analysis were performed similarly to Johnston, et al.^4^. Adapter trimmed sequence reads in FASTQ format were assessed for quality using FastQC (version 0.11.7) (http://www.bioinformatics.babraham.ac.uk/projects/fastqc/) and passed all the basic quality statistics. Reads were aligned to the UMD3.1 bovine reference genome and read counts were generated by converting aligned reads into counts per gene using the Spliced Transcripts Alignment to a Reference (STAR) aligner (version 2.6.1b)^11^.

Differential gene expression was determined using the R (R version 3.6.1 (2019-07-05)^12^ Bioconductor package EdgeR (version 3.28.0) which models data as a negative binomial distribution to account for biological and technical variation^13^. To remove lowly expressed genes, any genes with less than one count per million in at least six of the samples, were removed from the analysis. Data were normalised across libraries using the trimmed mean of M-values normalisation method^14^ and dispersion was estimated using the quantile-adjusted conditional maximum likelihood (qCML) common dispersion and the qCML tagwise dispersion. Exact tests were used for the detection of DE genes between BRSV challenged and control calves, considering genes with a Benjamini–Hochberg false discovery rate (FDR) of 10% and a fold change of ≥ 2 to be DE.

### Pathway and gene ontology analysis

The DEG between BRSV challenged and control calves, with an FDR of 10% and a fold change of > 2, were input into ClusterProfiler (version v3.14.0)^15^ in R [version 3.6.1 (2019-07-05)]^12^, for Database for Annotation, Visualization and Integrated Discovery (DAVID)^16,17^ pathway and GO analysis using the “EnrichDAVID” function. The annotation types interrogated included: “GOTERM_BP_ALL”, “GOTERM_MF_ALL”, “GOTERM_CC_ALL” and “KEGG_PATHWAY”. Pathways and GO terms from the DAVID ClusterProfiler analyses were considered enriched at a P value of less than 0.05 and an FDR of 5%.

## Results

Clinical scores and lung pathology results have been previously described^4^. Briefly, there were no significant differences in clinical scores between the BRSV challenged calves and the control calves at any of the time-points and there were no differences in lung scores between the BRSV challenged calves and the controls. However, Fisher’s exact tests indicated that BRSV challenged calves had a higher probability of having a lesioned lung compared with the control calves.

**Figure 1:**
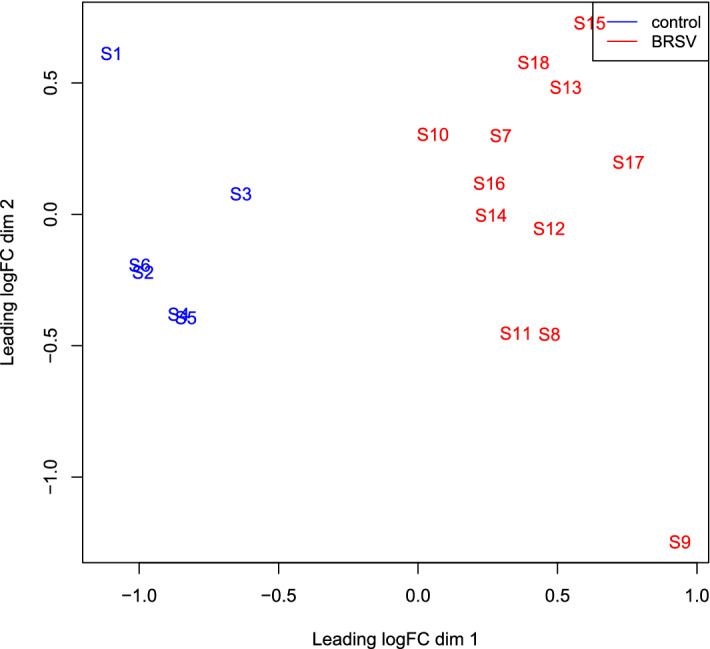
An MDS plot generated in EdgeR^12,13^ illustrating the similarity of the samples based on the first two principal components of gene expression covariance matrix among individuals. Samples from BRSV challenged calves are coloured in red and samples from control calves are coloured in blue. The numbers (1–18) refer to the calf ID and the letter S in front of the numbers refers to the word “sample”.

An average of (mean ± SD) 41,242,289 ± 5,623,575 sequenced fragments were received in FASTQ format. Following quality and adapter trimming, 40,678,972 ± 5,549,466 sequence reads remained and 91.9% ± 1.29% were uniquely mapped to the UMD3.1 bovine reference genome. An MDS plot produced by EdgeR displayed a clear separation between BRSV challenged and control calves based on whole blood gene expression changes, despite an observed mild clinical manifestation of the disease (Fig. 1).

There were 281 differentially expressed (DE) genes (*p* < 0.05, FDR < 0.1, fold change > 2) between the BRSV challenged and control calves (Supplementary Table S1). The most up-regulated genes in whole blood in response to BRSV were *CCL8, SLCO2B1, ADM* and *IFI27,* which were all up-regulated by at least 15 fold. The genes with the greatest transcriptional down-regulation in whole blood due to the BRSV challenge were *COL1A2* and *COL1A1*, which were down-regulated by at least 17 fold.

**Figure 2:**
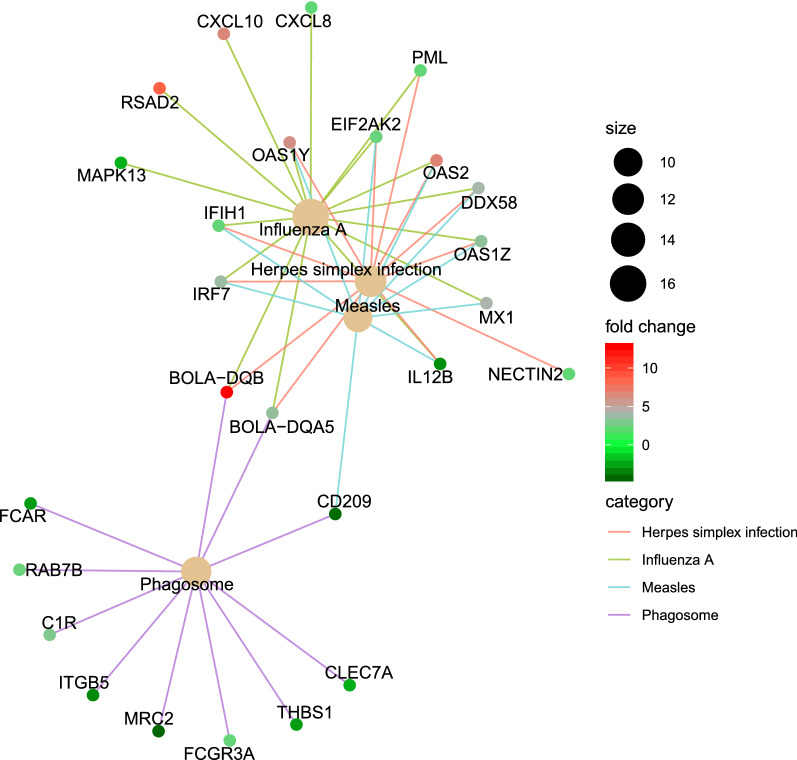
Cnet plot of enriched KEGG pathways (*P* < 0.05, FDR < 0.05). This plot was produced in ClusterProfiler^12,15^ based on the results of the “EnrichDAVID”^16,17^ function. Category = the enriched KEGG pathway. Size = the number of differentially expressed genes which belong to the enriched KEGG pathway. Fold change = the fold change difference between the BRSV challenged calves relative to the control calves.

**Figure 3:**
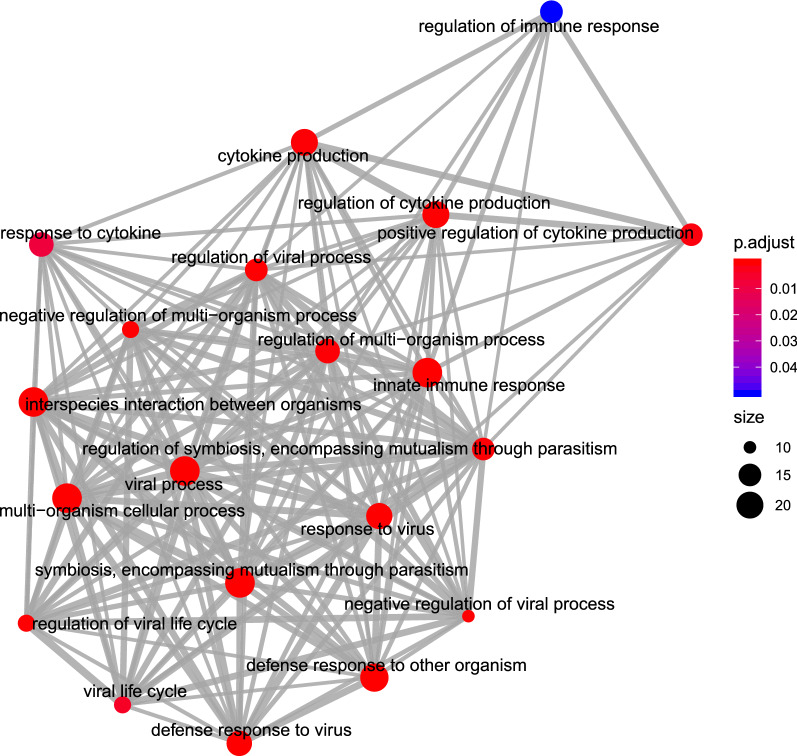
Emap plot of enriched “Biological Process” gene ontology terms (*P* < 0.05, FDR < 0.05). This plot was produced in ClusterProfiler^12,15^ based on the results of the “EnrichDAVID”^16,17^ function. p.adjust = the Benjamini–Hochberg adjusted P-value for the enriched ontology term. Size = the number of differentially expressed genes which belong to the enriched gene ontology term.

There were four enriched KEGG pathways among the DE genes in whole blood between the BRSV challenged and control calves (*P* < 0.05, FDR < 0.05); bta05164 “Influenza A”, bta05168, “Herpes simplex infection”, bta04145 “Phagosome” and bta05162 “Measles” (Fig. 2). There were twenty enriched gene ontology “biological process” terms (*P* < 0.05, FDR < 0.05), and the majority of the ontology terms were associated with the immune response to viral infection, including “innate immune response”, “defense response to virus”, “viral process”, “negative regulation of viral process”, “regulation of viral life cycle”, and “cytokine production” (Fig. 3).

## Discussion

This is the first study to identify mRNA biomarkers and molecular pathways of BRSV in circulating whole blood samples from experimentally challenged calves. Previously, several genes have been shown to be transcriptionally altered in whole blood at feedlot entry and during BRD incidents compared to when healthy and market ready^18^. However, the specific viral or bacterial agents involved in the BRD incidents were not identified in this study. Other studies have identified BRD biomarkers such as phenylalanine, lactate, hydroxybutyrate, tyrosine, citrate and leucine, in the blood of feedlot cattle using blood 1H NMR metabolomics, which could also be used in the diagnosis of BRD^19^. Furthermore, differences in systemic gene expression between beef cattle at feedlot arrival that go on to develop BRD and those who remain BRD free, were identified and can potentially act as biomarkers of BRD disease risk in feedlot cattle^8^. There is increasing evidence that the interactions that characterize disease and host immune responses can be examined via RNA-Seq, and identify relationships between host gene expression and BRD outcome^8^. The present study has identified genes which are DE in response to a specific viral cause of BRD, BRSV, in artificially-reared dairy calves.

Several genes were up-regulated between 15 and 22 fold (*CCL8, SLCO2B1, ADM*, *IFI27*), and two genes (*COL1A2, COL1A1*) were down-regulated between 17 and 24 fold, in BRSV challenged relative to control heathy calves. *CCL8* is a chemokine which attracts monocytes, lymphocytes, basophils and eosinophils to the site of infection and plays a role in the host’s proinflammtory response. *CCL8* has also been observed to be transcriptionally increased in bronchoalveolar lavage from calves experimentally challenged with a novel viral member of the BRDC, influenza D^20^. Furthermore, *CCL8* concentrations were 22.8 fold greater in idiopathic pulmonary fibrosis fibroblasts relative to control lung tissue fibroblasts and were additionally higher in bronchoalveolar lavage fluid from idiopathic pulmonary fibrosis patients compared with controls^21^. The *SLCO2B1* gene is a member of the organic anion transporting polypeptide family and is involved in the cellular uptake and transport of compounds^22^ and the *ADM* gene encodes a vasodilator protein which plays a role in the inflammatory response^23^. The *IFI27* gene is also involved in the inflammatory response including type-I interferon-induced apoptosis and the host antiviral response to hepatitis C^24^. COL1A2 and COL1A1 are members of the collagen family which provide structural support to the extracellular matrix, and their expression levels have previously been associated with several human cancers^25^. Expression changes in blood induced by BRSV in these genes may be validated in larger populations and have potential to act as diagnostic biomarkers of BRSV infection in whole blood.

The top enriched KEGG pathways and gene ontology terms were associated with viral infection and included “Influenza A”, “defense response to virus”, “regulation of viral life cycle” and “innate immune response”. Highly DE genes involved in these pathways may be beneficial for the diagnosis of subclinical BRD from blood samples. They encompass genes involved in the anti-viral interferon response, including *ISG15, IL12B, ISG20, OAS1Z, DDX58, IFIH1, OAS2, DHX58, PRF1, EIF2AK2, OAS1Y, IFIT2, IFIT5, IFI6, IFITM5, IFITM3, IFI44, IFI44L, IFI27, MX1, MX2, CXCL10 and RSAD2*. Several of these genes, *IFI6, ISG15, MX1,* and *OAS2* were identified as driver genes of interferon signalling and biomarkers for prediction at feedlot entry which beef cattle will go on to develop BRD^18^. Interferon-stimulated genes, including *IFI44, IFI6, IFIT2, ISG15, MX1, MX2,* and *RSAD2* have been postulated to interfere with peste des petits ruminants virus (which is a member of the same family as BRSV, *Paramyxoviridae*) replication in cattle and their up-regulation in cattle relative to goats’ peripheral blood mononuclear cells appears to prevent clinical illness from this virus in cattle^26^. Furthermore, these genes play important conserved roles in the host response to viral infections in different species, including the bovine^27^. *IL12B* and *CXCL10* were transcriptionally up-regulated due to bovine viral diarrhoea 2 infection in goats’ peripheral mononuclear cells^28^. The *ISG15* gene is strongly induced and the protein is rapidly produced in response to viral infections and it has been shown to interfere with replication of several viruses, including Influenzas A and B, and to modulate host immunity^27,29^. Similarly, ISG20 interferes with replication of viruses, including the hepatitis C and A viruses and bovine viral diarrhoea virus^30^. The IFIT family of proteins, of which several gene members were up-regulated in the blood of the BRSV challenged relative to control calves, *IFIT2* and *IFIT5,* also play a role in the inhibition of viral replication through binding of viral proteins and RNAs^31^. The IFITM proteins, of which the *IFITM3* and *IFITM5* genes were transcriptionally increased, are involved in the restriction of the entry of enveloped viruses into the host cell^32^. Additionally, this study, consistent with previous studies^4,6,8,18^, provides further evidence of a Th1 skewed, interferon dominated, transcriptional immune response to BRSV, due to the systemic up-regulation of interferon stimulated genes. Similarly, human RSV also induces interferon genes in infants and a more intense interferon response is observed in patients with milder disease symptoms^33^, which is consistent with this bovine study as despite substantial changes in gene expression, the BRSV infected calves only presented with a mild clinical response.

It is interesting that viral specific gene expression was systemically altered in the present study despite the lack of an observation of a clinical response to the BRSV infection. This is consistent with the observation that many of the same genes are DE and same functions are enriched in beef cattle at feedlot entry (in the subclinical state) as when BRD is identified in these animals, relative to the healthy market ready state^18^. This indicates that these genes may be useful potential biomarkers of subclinical BRD and therefore may lead to the earlier identification and treatment of animals and subsequent reduction in severe disease bouts and the necessity for profuse antimicrobial treatments. As this study has confirmed that these gene-based biomarkers of BRSV are present in whole blood in addition to respiratory associated tissues, there is potential for the development of a PCR or sequencing based non-invasive diagnostic test for BRSV, even at the subclinical stage, that can be performed on live animals through acquisition of a routine blood sample.

## Supplementary Information

Supplementary Information.

## Data Availability

All sequence data produced in this study has been deposited to NCBI GEO repository and are available through series accession number GSE152959.
